# Selective Modulation of the GluN2B/C/D Containing *N*‑Methyl‑d‑Aspartate Receptors: A New Frontier in Targeted Neurotherapeutics

**DOI:** 10.1021/acsmedchemlett.5c00365

**Published:** 2025-06-26

**Authors:** Yinlong Li, Steven H. Liang

**Affiliations:** Department of Radiology and Imaging Sciences, 1371 Emory University, 1364 Clifton Road, Atlanta, Georgia 30322, United States

**Keywords:** NMDARs, GluN2B/C/D subunits, Positive allosteric modulators (PAMs), Structure−activity relationship (SAR), Neurological disorders

## Abstract

*N*-methyl-d-aspartate receptors (NMDARs) are a class of ionotropic glutamate receptors that mediate synaptic plasticity and excitatory neurotransmission throughout the central nervous system (CNS). Dysregulation of NMDAR function has been implicated in multiple neurological disorders such as autism, schizophrenia, and depression. Thus, NMDARs are considered crucial therapeutic targets, and extensive studies have focused on the development of NMDAR modulators. Positive allosteric modulators (PAMs) represent a promising approach to regulate NMDARs hypofunction; however, the availability of subunit-selective PAMs remains limited. A recent study has identified a series of GluN2B/C/D-biased PAMs with approximately 20-fold increased potency and high subunit selectivity through structure–activity relationship (SAR) optimization, which provides valuable insights for NMDARs-targeted drug development.

## Graphical abstract

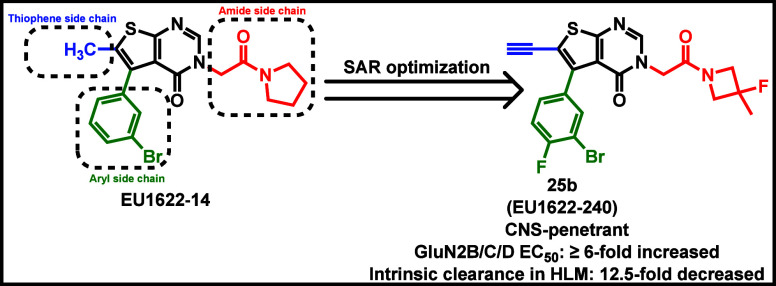

Ionotropic glutamate receptors (iGluRs) are ligand-gated cation channels that regulate excitatory neurotransmission in the central nervous system (CNS).^1–5^ Based on pharmacological properties, iGluRs are classified into α-amino-3-hydroxy-5-methyl-4-isoxasolepropionic acid receptors (AMPARs),^6^ kainate receptors,^7^ and *N*-methyl-d-aspartate receptors (NMDARs).^8,9^ NMDARs exhibit unique voltage-dependent blockade mediated by physiological levels of extracellular magnesium (Mg^2+^) at resting membrane potential.^10^ Upon coactivation by the excitatory neurotransmitters (glycine and glutamate), NMDARs become highly permeable to calcium (Ca^2+^), sodium (Na^+^), and potassium (K^+^) ions.^11^ This process triggers critical downstream signaling cascades that are crucial for synaptic plasticity, learning, and memory.^12,13^ NMDARs are heterotetrameric receptor complexes composed of GluN1, GluN2 (GluN2A-D), and GluN3 (GluN3A-B) subunits.^14–16^ The dysregulation of NMDARs has been linked to various neurological and psychiatric disorders, including autism,^17^ schizophrenia^18^ and Alzheimer’s disease (AD).^19^ The development of subunit-selective modulators capable of enhancing or inhibiting specific NMDAR subtypes remains a critical research focus in neuroscience.^20,21^ The spatiotemporal expression patterns of GluN2 subunits confer distinct functional characteristics to NMDARs across different brain regions.^22,23^ These subunits critically modulate key receptor properties, including agonist potency, synaptic-like response time course, ion permeability, channel open probability, and voltage-dependent Mg^2+^ block. Therefore, significant efforts have been focused on developing GluN2 subunit-selective small molecules to precisely target distinct neural circuits and attain specific desired therapeutic benefits.^24,25^

Several GluN2-targeted positive allosteric modulators (PAMs) have been developed to enhance NMDAR function with subunit specificity.^26,27^ Compounds such as Plazinemdor (EC_50_ range 0.120–0.220 μM),^28^ SAGE-718 (EC_50_ range 0.079–0.37 μM),^29^ and GNE-9278 (EC_50_ range 3–16 μM)^30^ exhibit broad activity across GluN2A–2D subunits, and some have progressed to clinical evaluation. In contrast, Zelquistinel (EC_50_ range 9.7–9.9 μM) demonstrates selectivity for GluN2B/2C subunits and has shown antidepressant-like effects,^31^ while GNE-0723 is a GluN2A-selective PAM with cognitive-enhancing properties (EC_50_: 0.021 μM).^32^ These modulators represent valuable lead compounds for the treatment of neuropsychiatric and neurodegenerative disorders. In 2020, a thienopyrimidinone-based GluN2A-2D PAM EU1622-14 was identified through a fluorescence-based screen.^33^ EU1622-14 increases agonist potency, prolongs the receptor deactivation time course, and raises channel open probability. At the same time, it reduces single-channel conductance and lowers calcium permeability, which may help preserve receptor efficacy while minimizing calcium-mediated excitotoxicity. Structural characterization has identified an allosteric binding pocket localized at the interface of the GluN2 pre-M1 linker and M3 transmembrane helix. The distinct thiophene, aryl, and amide side chains of EU1622-14 offer opportunities for structure–activity relationship (SAR) optimization to further enhance potency, selectivity, and metabolic stability (Figure 1).

**Figure 1:**
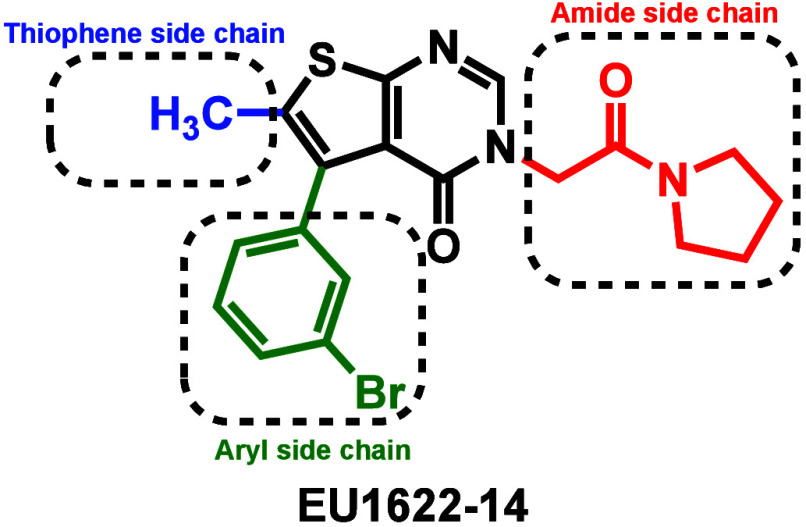
EU1622-14 structure with highlighted SAR optimization sites. The figure was adapted from ref 34. Copyright 2025 American Chemical Society.

A recent advancement in the development of EU1622-14 analogues utilized a pharmacophore-based design strategy by systematically modifying the three side chains extending from the thienopyrimidinone core (Figure 2).^34^ NMDAR PAM activity was assessed using two-electrode voltage clamp (TEVC) recordings in *Xenopus laevis* oocytes. Initial exploration focused on the amide side chain, exploring a range of substituted pyrrolidines and azetidines bearing alkyl and fluorine groups, as well as stereochemical preferences. Among these, compound **6m**, featuring a 3-fluoro-3-methyl disubstitution showed significantly improved potency, with doubling concentrations of 0.6 μM (GluN2B), 0.5 μM (GluN2C), and 0.5 μM (GluN2D). Guided by prior SAR indicating a preference for substitution at the 3-position of the aryl ring, a series of 3-position and 3,4-disubstituted analogues, such as Cl, CF_3_, OCF_3_, and CN, were synthesized and evaluated. SAR studies revealed that lipophilic halogen substitutions, particularly 3-chloro-4-fluoro and 3,4-dichloro, enhanced potency across GluN2B/C/D subunits, but were associated with reduced solubility. The 3-bromo-4-fluoro analog (**12j**) demonstrated an optimal balance, which maintains robust potency (EC_50_: 0.33–1.1 μM) with moderate lipophilicity (cLogP 3.9). Building on the optimized 3-bromo-4-fluoro aryl scaffold of **12j**, the authors further modified thiophene side chains. Modifications such as reducing the alkyl chain to ethyl or hydrogen, or incorporating a CF_3_ group, led to diminished potency across all GluN2 subtypes. In contrast, introducing an alkyne moiety yielded compound **25b** (EU1622-240), which maintained EC_50_ values below 1 μM and significantly improved doubling concentrations, less than 350 nM, at GluN2B/2C/2D. These results highlight **25b** as a promising lead candidate for further evaluation.

**Figure 2:**
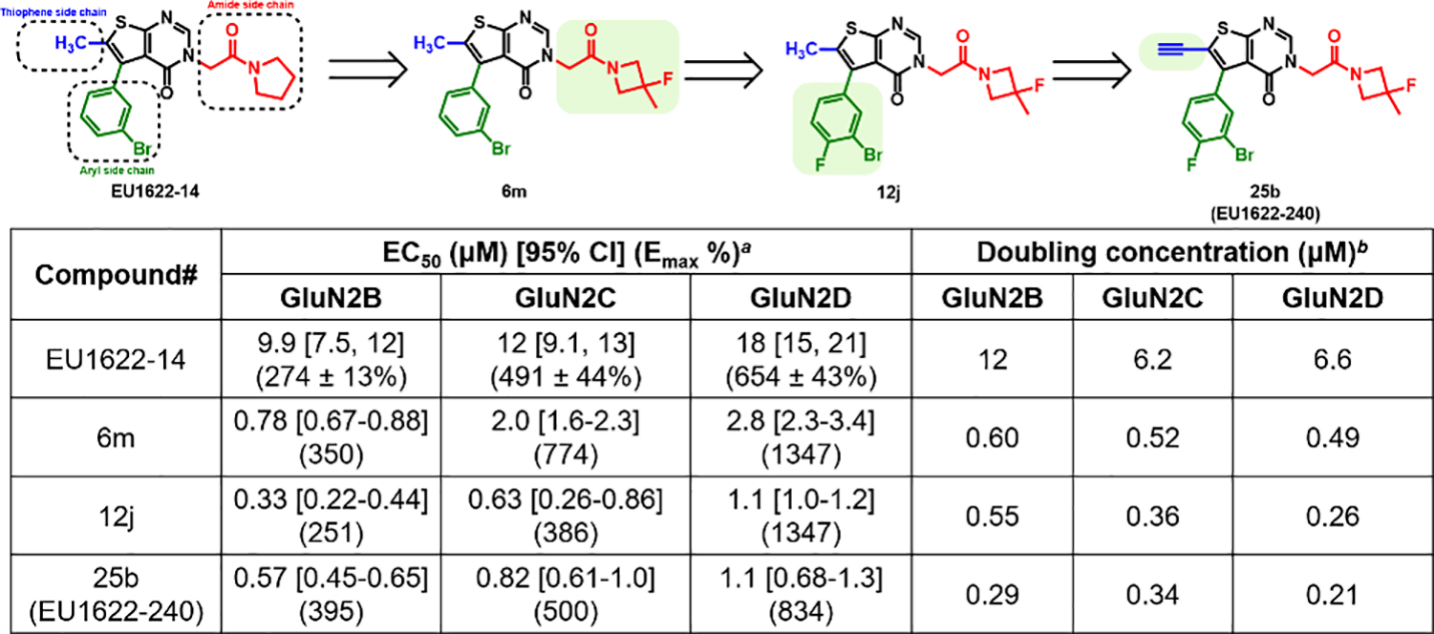
SAR optimization of EU1622-14. *^a^* EC_50_ values with 95% confidence intervals (CI) were calculated from log-transformed dose–response curves, with the maximum response (*E*_max_) normalized to baseline current elicited with 100 μM glutamate and 30 μM glycine. *^b^* The doubling concentrations were determined through the Hill equation fitting of concentration–response data or estimated by interpolation when response plateaus were not observed. The data was adapted from ref 34. Copyright 2025 American Chemical Society.

As shown in Figure 3, the drug metabolism and pharmacokinetics (DMPK) properties of **25b** were evaluated. Kinetic aqueous solubility in PBS (pH 7.3) was 40 ± 20 μM, which is lower than EU1622-14 (100 μM) but sufficient for *in vitro* assays. **25b** exhibited excellent plasma stability across human, mouse, and rat species with *t*_1/2_ > 4 h in all cases. Compared with EU1622-14 which showed high intrinsic clearance in human liver microsomes (HLMs) (Cl_int_ = 50 μL/min/mg), **25b** demonstrated enhanced metabolic stability with lower clearance (Cl_int_ = 4.8 μL/min/). In the MDCK-MDR1 assay, **25b** exhibited high in vitro permeability (P_app_ A-B = 15.26 × 10^–6^ cm/s) with mild P-gp efflux (ER = 1.61), suggesting favorable CNS penetration. Additionally, **25b** exhibited good oral bioavailability (58%) and moderate CNS exposure (K_p_ = 0.6).

**Figure 3:**
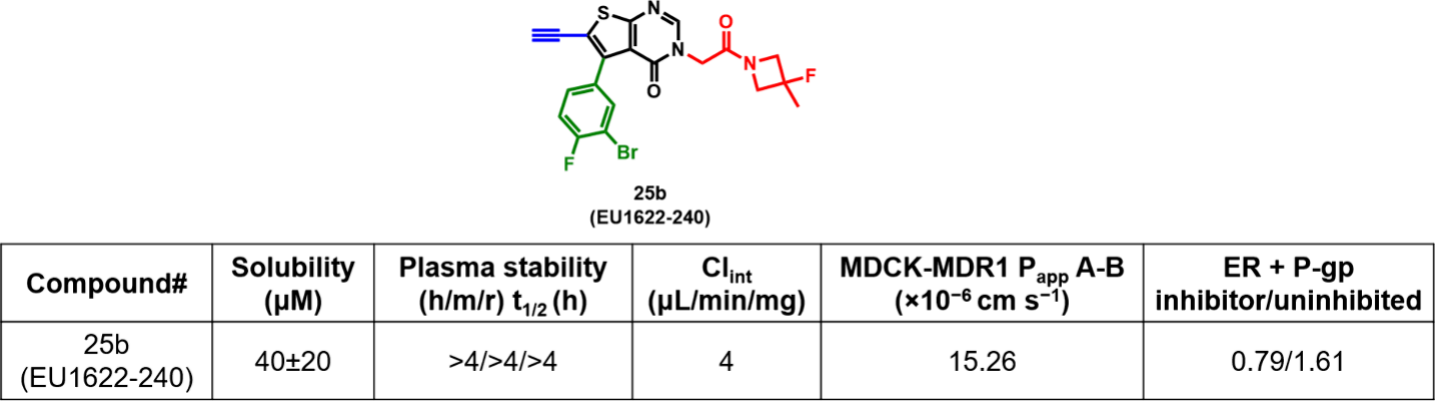
DMPK properties of **25b** (EU1622-240). Intrinsic clearance (Cl_int_) was measured in HLMs. The data was adapted from ref 34.

To evaluate the in vivo effects of **25b**, Open Field and Light/Dark Box tests were conducted in mice (Figure 4). Compound **25b** was administered intraperitoneally at doses of 5 mg/kg or 10 mg/kg. Mice treated with 10 mg/kg of **25b** exhibited reduced avoidance behavior in the Open Field test, showing significantly increased percentage of distance in the center of the field (30.2 ± 3.03%) compared to vehicle-treated controls (22.9 ± 2.89%) and those treated with 5 mg/kg **25b** (20.8 ± 2.09%). Similarly, in the Light/Dark Box test, the 10 mg/kg group showed significant increases in both the percentage of distance traveled and the time spent in the light chamber. These findings demonstrate dose-dependent efficacy of **25b** in reducing avoidance behaviors, which is consistent with potential anxiolytic or pro-exploratory effects.

**Figure 4:**
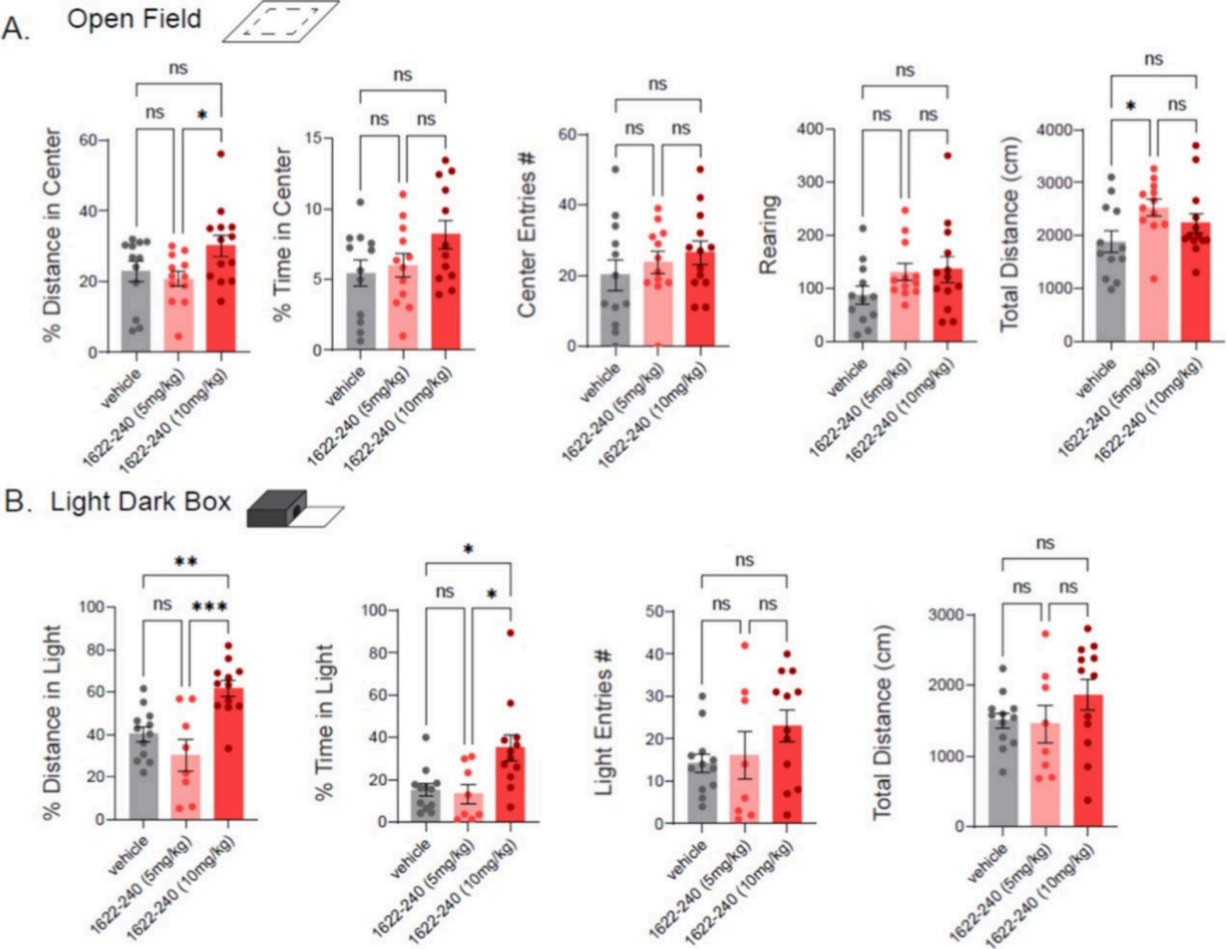
NMDAR potentiation modulates behavior. (A) Summary of mouse behavior in the open field test conducted for 10 min, 1 h after intraperitoneal administration of **25b** at 5 or 10 mg/kg. (B) Summary of behavior in the light/dark box test under the same treatment conditions and time frame. Mice spent more time and traveled greater distances in the light chamber is consistent with reduced anxiety-like behavior, potentially indicative of enhanced interneuron function. *p < 0.05, one-way ANOVA with Tukey’s test. The data was adapted from ref 34. Copyright 2025 American Chemical Society.

Given its promising pharmacological profile, compound **25b** (EU1622-240) has emerged as the lead compound from a series of EU1622-14 analogues following an extensive SAR screening. Future studies should prioritize elucidating its molecular mechanism of action, including GluN2A-D subtype specificity and brain region–specific effects. Moreover, behavioral assessments to chronic stress and social interaction models would offer a more comprehensive understanding of EU1622-240s therapeutic potential. While continuous medicinal chemistry efforts to enhance pharmacological properties is critical for advancing EU1622-240 to clinical development, molecular imaging techniques such as positron emission tomography (PET),^35,36^ could accelerate clinical translation by providing valuable insights into its brain distribution and receptor occupancy.

## Notes

No unexpected or unusually high safety hazards were encountered.

## Abbreviations

NMDARs: *N*-methyl-d-aspartate receptors
CNS: central nervous system
PAMs: positive allosteric modulators
SAR: structure–activity relationship
iGluRs: ionotropic glutamate receptors
AMPARs: α-amino-3-hydroxy-5-methyl-4-isoxasolepropionic acid receptors
Mg^2+^: magnesium
Ca^2+^: calcium
Na^+^: sodium
K^+^: potassium
AD: Alzheimer’s disease
TEVC: two-electrode voltage clamp
CI: confidence intervals
*E*_max_: maximum response
DMPK: drug metabolism and pharmacokinetics
HLMs: human liver microsomes
Cl_int_: intrinsic clearance
PET: positron emission tomography
